# Retrospective motion correction through multi‐average k‐space data elimination (REMAKE) for free‐breathing cardiac cine imaging

**DOI:** 10.1002/mrm.29613

**Published:** 2023-02-10

**Authors:** Alexander Paul Neofytou, Radhouene Neji, Grzegorz Tomasz Kowalik, Ronald Mooiweer, James Wong, Anastasia Fotaki, Joana Ferreira, Carl Evans, Filippo Bosio, Nabila Mughal, Reza Razavi, Kuberan Pushparajah, Sébastien Roujol

**Affiliations:** ^1^ School of Biomedical Engineering and Imaging Sciences, Faculty of Life Sciences and Medicine King's College London London UK; ^2^ MR Research Collaborations Siemens Healthcare Limited Newton House, Sir William Siemens Square, Frimley, Camberley Surrey UK; ^3^ Department of Paediatric Cardiology Evelina London Children's Hospital London UK

**Keywords:** 2D cine, cardiac MRI, free‐breathing, retrospective respiratory motion correction

## Abstract

**Purpose:**

To develop a motion‐robust reconstruction technique for free‐breathing cine imaging with multiple averages.

**Method:**

Retrospective motion correction through multiple average k‐space data elimination (REMAKE) was developed using iterative removal of k‐space segments (from individual k‐space samples) that contribute most to motion corruption while combining any remaining segments across multiple signal averages. A variant of REMAKE, termed REMAKE+, was developed to address any losses in SNR due to k‐space information removal. With REMAKE+, multiple reconstructions using different initial conditions were performed, co‐registered, and averaged. Both techniques were validated against clinical “standard” signal averaging reconstruction in a static phantom (with simulated motion) and 15 patients undergoing free‐breathing cine imaging with multiple averages. Quantitative analysis of myocardial sharpness, blood/myocardial SNR, myocardial‐blood contrast‐to‐noise ratio (CNR), as well as subjective assessment of image quality and rate of diagnostic quality images were performed.

**Results:**

In phantom, motion artifacts using “standard” (RMS error [RMSE]: 2.2 ± 0.5) were substantially reduced using REMAKE/REMAKE+ (RMSE: 1.5 ± 0.4/1.0 ± 0.4, *p* < 0.01). In patients, REMAKE/REMAKE+ led to higher myocardial sharpness (0.79 ± 0.09/0.79 ± 0.1 vs. 0.74 ± 0.12 for “standard”, *p* = 0.004/0.04), higher image quality (1.8 ± 0.2/1.9 ± 0.2 vs. 1.6 ± 0.4 for “standard”, *p* = 0.02/0.008), and a higher rate of diagnostic quality images (99%/100% vs. 94% for “standard”). Blood/myocardial SNR for “standard” (94 ± 30/33 ± 10) was higher vs. REMAKE (80 ± 25/28 ± 8, *p* = 0.002/0.005) and tended to be lower vs. REMAKE+ (105 ± 33/36 ± 12, *p* = 0.02/0.06). Myocardial‐blood CNR for “standard” (61 ± 22) was higher vs. REMAKE (53 ± 19, *p* = 0.003) and lower vs. REMAKE+ (69 ± 24, *p* = 0.007).

**Conclusions:**

Compared to “standard” signal averaging reconstruction, REMAKE and REMAKE+ provide improved myocardial sharpness, image quality, and rate of diagnostic quality images.

## Abstract 2

Click here for author‐reader discussions

## 1 INTRODUCTION

Assessment of cardiac anatomy and function is important for the diagnosis and management of a variety of cardiac diseases.^1^, ^2^, ^3^, ^4^ Cine MRI is the gold standard technique for the assessment of ventricular anatomical and functional parameters (e.g., ejection fraction, volume, and mass) and is used in the vast majority of cardiac MRI protocols due to its high accuracy and reproducibility.^5^, ^6^, ^7^, ^8^, ^9^

Cine acquisition typically uses a balanced SSFP (bSSFP) sequence, providing excellent blood‐myocardial contrast and high spatio‐temporal resolution. A stack (˜12–14 slices) of cine images in the short axis orientation (SAX) is typically acquired to achieve full left ventricular (LV) coverage. High spatio‐temporal resolution is achieved using segmented acquisition. Images are typically acquired under breath‐hold conditions to avoid respiratory motion artifacts (e.g., blurring). Each breath‐hold is about ˜10–12 s long, during which one to two slices are acquired. Multiple breath‐holds are thus required for full LV coverage.^10^

Such protocols can be challenging for patients unable to tolerate multiple breath‐holds. Several approaches have been proposed for single breath‐hold CINE protocols using highly accelerated schemes based on compressed sensing^11^, ^12^ or deep learning‐based reconstructions.^13^ However, all these aforementioned approaches require patient cooperation, which can be difficult for those who cannot perform breath‐hold maneuvers, particularly young children or those with impaired cognitive function or limited breath‐hold capabilities. Respiratory motion corrupted images can make it difficult or impossible to accurately delineate the ventricular volumes necessary for LV analysis.

Alternatively, a variety of free‐breathing techniques have been proposed. Respiratory gating enables the acquisition (prospective gating) or reconstruction (retrospective gating) of data at a given respiratory position (i.e., expiration). To track the respiratory motion during the acquisition process, the use of external respiratory sensors such as a respiratory bellow or Pilot tone has been proposed.^14^, ^15^ However, these sensors do not measure directly the displacement of the heart, which limits the automatic definition of an optimal gating window and can additionally require the use of a calibration scan. Diaphragmatic navigators^16^ and image navigators directly positioned on the heart^17^ can be used to track the respiratory motion of the heart. However, the use of these navigators requires the interruption of the continuous cine acquisition, leading to temporal information gaps and potential disruption of the steady state signal. To address this issue, a variety of self‐gating methods have been proposed using non‐cartesian k‐space trajectories.^18^, ^19^, ^20^, ^21^, ^22^, ^23^, ^24^, ^25^ However, these methods require lengthy reconstruction times and limit the use of conventional reconstruction techniques. Overall, most of these techniques rely on additional hardware or customized pulse sequences, which are not currently available across all scanner types/manufacturers.

Retrospective respiratory motion correction methods have also been proposed.^20^, ^21^, ^22^, ^23^, ^24^, ^25^, ^26^, ^27^, ^28^, ^29^, ^30^, ^31^, ^32^ Free‐breathing continuous imaging methods combined with retrospective binning according to respiratory and cardiac motion states have been presented^20^, ^21^, ^22^, ^23^, ^25^, ^26^, ^27^, ^28^, ^29^; however, these techniques are generally associated with prolonged scan and reconstruction times.^20^, ^21^, ^22^, ^23^, ^24^, ^25^, ^26^, ^27^, ^28^, ^29^ Advanced averaging methods have been proposed in this context using single‐shot acquisition with multiple NSAs and have been demonstrated for coronary imaging,^33^ late gadolinium enhancement imaging,^34^ as well as CINE imaging.^26^, ^28^, ^30^, ^32^ Advanced averaging is used to compensate for inter‐scan motion (i.e., motion between single‐shot images) by only averaging a subset of images acquired at a consistent breathing position and/or by applying image registration to compensate for breathing motion between images. This initial approach however resulted in lower spatio‐temporal resolution, associated with single‐shot imaging.^32^ This approach was extended by combining prolonged scanning (60 s/slice) and retrospective binning according to cardiac motion states to generate images with higher spatio‐temporal resolution.^26^, ^28^ Scan time per slice was later reduced using non‐linear iterative reconstruction with temporal regularization^30^; however, this technique remained associated with long reconstruction times and enlarged temporal footprint due to temporal regularization. Finally, AI‐based retrospective correction of respiratory motion for free‐breathing 2D cine imaging has also been proposed, although residual motion artifacts were observed, particularly in systolic phases.^31^

Free‐breathing segmented cine acquisition with multiple number of signal averages (typically NSA = 3) is an alternative strategy for patients unable to breath‐hold^7^, ^10^ and is widely available in most scanners. This technique enables the prescription of high spatio‐temporal resolution, comparable to standard breath‐hold acquisition and requires no additional hardware or respiratory gating. The consequence of having no gating allows for k‐space segments to be acquired at different respiratory phases which can result in degradation of the effective resolution and reduced sharpness.

In this work, we sought to develop a motion‐robust reconstruction technique using retrospective motion correction through multi‐average k‐space data elimination (REMAKE) for free‐breathing cine imaging. The basis of REMAKE relies on the iterative removal of k‐space segments (from individual k‐space samples) that contribute most to motion corruption (or blurring) while combining any remaining segments across multiple signal averages. Multiple reconstructions are performed using different initial conditions (REMAKE+), which are co‐registered using non‐rigid image registration and averaged to conserve the SNR. REMAKE is fully automated (i.e., no user input required for drawing regions of interest [ROIs] or defining respiratory gating windows), does not require any additional hardware (e.g., the use of respiratory sensors) or sequence modifications and does not affect the temporal footprint of the sequence. The method is validated in‐vitro in phantom and in‐vivo using a free‐breathing cine protocol evaluated in 15 patients. A subset of the data presented here was presented at the 2022 ISMRM conference.^35^

## 2 METHODS

### 2.1 Proposed REMAKE and REMAKE+ reconstruction and implementation

**FIGURE 1:**
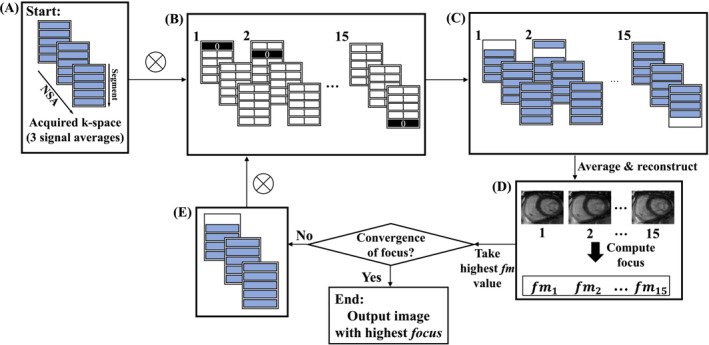
Proposed algorithm for the iterative removal of k‐space segments. In this example, there are five segments per k‐space matrix and NSA = 3, leading to 15 possible k‐space segment rejections. The algorithm begins with the input of the acquired k‐space matrices used to reconstruct one image. (A) This is the initial k‐space configuration. The element‐wise multiplication (represented here by ⊗) of the inputted k‐space matrices with a binary multidimensional array (B) is performed to compute a set of all possible ways one segment can be removed (C). (D) All resulting k‐space segment configurations are then averaged and reconstructed into the image domain, where the focus measure is computed for all reconstructed images. (E) The k‐space segment configuration that outputs the highest image focus measure, replaces the initial k‐space configuration. The algorithm will then repeat to find the next segment removal that increases the image focus when removed until convergence of the image focus is met.

**FIGURE 2:**
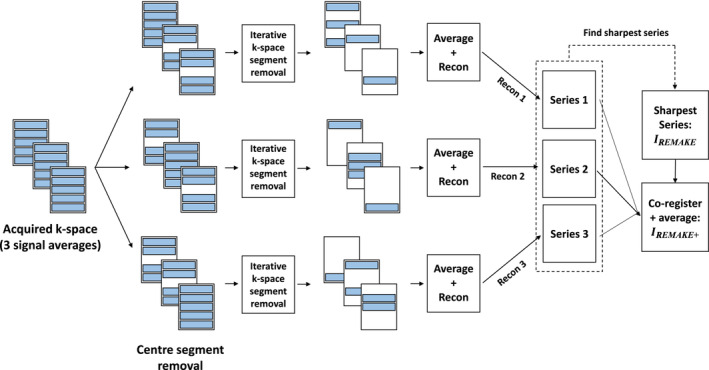
REMAKE and REMAKE+ reconstructions. The reconstruction pipeline depicted is applied to each acquired k‐space data with three signal averages (i.e., each slice and cardiac phase) to create three unique reconstructed images from three unique initial k‐space configurations (removal of two of three center segments). On a slice‐by‐slice basis, the generated image series with the highest mean focus (over cardiac phases) was labeled as the REMAKE image series, $I_{\text{REMAKE}}$. The three series are then co‐registered and averaged to generate the REMAKE+ image series, $I_{\text{REMAKE}+}$.

REMAKE and REMAKE+ are summarized in Figures 1, 2 for a typical acquisition with three signal averages (NSA = 3). The reconstruction aims to remove k‐space segments (from individual k‐space samples) responsible for motion corruption/blurring. This process is performed independently for each slice and each cardiac phase and can be formulated as follows:

(1) $$\underset{s}{\text{argmax}}\;f\left(R\;T_{s}\;k\right).$$

Here s is a set of removed k‐space segments across multiple NSAs, k represents the total measured k‐space data (including all segments and all NSAs), $T_{s}$ is the binary operator used to keep/discard specific segments, R applies the image reconstruction (averaging across multiple k‐space matrices followed by a Fourier Transform or more advanced techniques such as parallel imaging or compressed sensing reconstruction methods), and f is the focus measure function. A focus measure was used as a surrogate for quantifying the level of blurring or motion in the reconstructed images. It was assumed that as blurring reduces, edges in the images will appear sharper and therefore have a higher focus measure.

The energy of image gradient (GRAE) metric (see Eq. 2) was used to quantify the image focus. The GRAE is formulated as follows,

(2) $$\text{GRAE}=\frac{1}{\mathrm{MN}}\sum_{m=1}^{M}\sum_{n=1}^{N}{\left|\nabla I\left(m,n\right)\right|}^{2},$$

where I(m,n) is an image of dimensions M by N. The GRAE provides a pooled value of the gradient magnitude response,^36^ in both the phase and read direction, when applied to the image I(m,n). The metric was selected amongst several other focus metrics due to its superior performance in detecting motion corrupt k‐space data (please see Table S1 and Figure S1 for more details).

Expression (1) is maximized iteratively, where one segment is permanently removed at each iteration. For a given iteration, the effect of removing each k‐space segment (i.e., one k‐space segment from one NSA) on the focus measure of the corresponding reconstructed image is determined (Figures 1A–C). Each image is reconstructed from the average across the three NSAs of all remaining k‐space segments. The reconstructed image which outputs the highest focus measure (Figure 1D), allows for the identification of the segment to be permanently removed at that iteration (Figure 1E). The algorithm is repeated iteratively until convergence of the image focus is met. A condition was set across the three NSAs that at least one of the three samplings of an individual segment needed to be retained to ensure full desired k‐space sampling.

To mitigate the potential loss of SNR/CNR associated with the removal of k‐space segments, the described reconstruction is performed three times using different initial k‐space segment configurations (i.e., initial removal of two out of three central segments) to force the generation of three different images corresponding to potentially different respiratory motion states (Figure 2). On a slice‐by‐slice basis, the image series with the highest mean focus (measured across cardiac phases) was labeled as the REMAKE image series, $I_{\text{REMAKE}}$. The remaining two series were registered with $I_{\text{REMAKE}}$ using non‐rigid image registration.^37^ An average of the three series (the two registered +  $I_{\text{REMAKE}}$) was generated for the REMAKE+ reconstructed series, $I_{\text{REMAKE}+}$.

### 2.2 Evaluation

All imaging was performed using a 1.5T MR scanner (MAGNETOM Aera, Siemens Healthcare, Erlangen, Germany). Patient scanning was approved by the National Research Ethics Service (15/NS/0030), with written informed consent obtained from all participants. Images were generated offline in MATLAB (MathWorks, Natick MA, USA).

#### 2.2.1 Phantom study

An experiment was conducted in which motion corruption was simulated in a stationary phantom to compare ground truth (i.e., non simulated motion) reconstructions with REMAKE and REMAKE+. A single transverse slice through a T1MES phantom^38^ was acquired using bSSFP cine, with the following parameters: TE/TR = 1.2/2.8 ms (partial echo: 79%), flip angle = 52°, FOV = 265 × 350 mm^2^, voxel size = 2.2 × 1.6 mm^2^, slice thickness = 8 mm, bandwidth [BW] = 930 Hz/px, GRAPPA factor = 2, NSA = 3, temporal resolution = 25 ms, no. segments = 6, no. slices = 1, simulated heartrate = 60 bpm.

Motion corruption was retrospectively applied to each k‐space line as a translation in the phase‐encoding direction based on their corresponding acquisition time and the following respiratory motion model:

(3) $$D\left(t\right)=A\cos^{6}\left(\frac{\pi t}{T}\right),$$

where D(t) represents the respiratory displacement at time t, and A and T=2.5s control the maximum amplitude and period of the respiratory motion, as previously reported.^39^, ^40^ Reconstruction was then performed using three different techniques: “standard”, REMAKE, and REMAKE+. Different motion‐corrupted experiments were performed using Eq. (3) with different motion amplitudes (*A* = [2–12] mm, step size = 2 mm).

The RMS error (RMSE) was evaluated between the original motion‐free reconstructed images and each of the reconstructed images of the artificially corrupted datasets. The mean RMSE over all motion experiments is reported for each reconstruction technique. The mean (over all experiments) SNR between the uncorrupted dataset and the reconstructions of the corrupted data using “standard”, REMAKE, and REMAKE+ are also reported. SNR was determined by taking the ratio of the mean signal intensity contained within an ROI overlapping a tube to the standard deviation of the signal intensity values contained within an ROI drawn in air with no motion artifacts present. For each experiment, the mean SNR was determined over all tubes.

Since the simulated motion applied to each segment is known and by assuming that two of the three NSAs of each segment with the highest motion amplitude should be discarded, a comparison of actual segments removed versus predicted was investigated by looking at the accuracy of correctly identified segments that were discarded. The accuracy was defined as the ratio of the total number of correctly identified segments removed over the total number of predicted segments to remove.

#### 2.2.2 Patient study

A total of 15 patients (11 male and 4 female, age = 34 ± 16 y) referred for a clinical cardiac MRI examination were recruited for this study. A standard stack of SAX cine images was acquired under free breathing conditions using a segmented bSSFP sequence. Imaging parameters were as follows: TE/TR = 1.2/2.8 ms (partial echo: 79%), flip angle = 52°, FOV = 265 × 350 mm^2^, voxel size = 2.2 × 1.6 mm^2^, slice thickness = 8 mm, BW = 930 Hz/px, GRAPPA factor = 2, NSA = 3, temporal resolution = 20–40 ms, no. segments = 4–10, no. slices = 15. All data were exported offline and three reconstructed datasets were generated for each scan: (1) “standard” reconstruction ($I_{\text{standard}}$), (2) REMAKE reconstruction ($I_{\text{REMAKE}}$), and (3) REMAKE+ reconstruction ($I_{\text{REMAKE}+}$).

**FIGURE 3:**
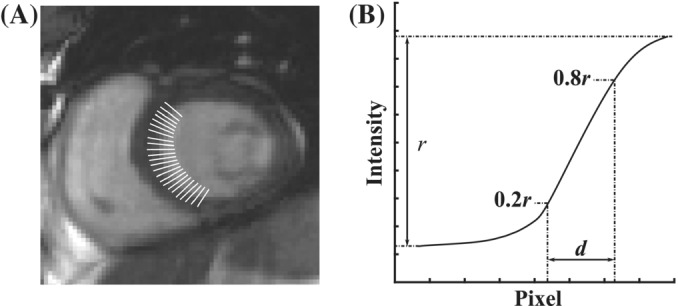
Illustration of sharpness measurement at the septal blood‐myocardium interface. (A) Line profiles drawn across the blood‐myocardium boundary. (B) Example of an intensity curve extracted from a line profile. The image sharpness is defined as the inverse of the pixel distance, 1/*d*, where *d*, is the pixel distance in which the intensity curve goes from 20% to 80% of the total intensity range, *r*.

Quantitative image analysis involving septal blood‐myocardium sharpness, SNR of blood and myocardium and their respective contrast‐to‐noise ratio (CNR) were also performed. For SNR and CNR measurements, noise was estimated as the standard deviation of the signal intensity values contained within an ROI drawn in the patient's lungs with no motion artifacts present. Septal blood‐myocardium sharpness was determined by drawing parallel curves, composed of closely spaced points, on either side of the septal blood‐myocardium boundary. Numerous line profiles perpendicular to the blood‐myocardium boundary were then generated (see Figure 3A). Each line profile produced an intensity curve as shown in Figure 3B. The image sharpness was defined as the inverse of the pixel distance (i.e., 1/*d*), where *d*, is the pixel distance in which the intensity curve goes from 20% to 80% of the total Intensity range, *r*, which is defined from the lowest intensity value (i.e., myocardium signal) to the highest intensity value (i.e., blood signal). The sharpness values computed for all line profiles are then averaged and reported.

Qualitative image assessment (0 = non‐diagnostic/severe motion artifacts, 1 = diagnostic (sub‐optimum image quality ‐ presence of motion artifacts/blurring not preventing interpretation/analysis of the images), 2 = diagnostic (excellent image quality ‐ no motion artifacts/no blurring/sharp myocardial edges)) was performed by consensus of two experienced cardiologists (J.W. and K.P., with respectively 8 and 10 y of cardiac MRI experience) on the three reconstruction techniques. Both readers were blinded from the clinical details of the patients and the reconstruction methods used, which were presented to them in a randomized order.

Further analysis of the fraction of segments (i.e., percentage of the total number of segments in a given slice or cardiac phase) removed was performed. This was investigated as a function of cardiac phase and slice through the SAX stack as well as comparing the fraction of segments removed against subjective scores given (0, 1 or 2) and SAX level (Base, Mid, Apex). Please refer to “In‐vivo analysis of discarded segments” and Figure S2 for more details.

### 2.3 Statistical analysis

Results for the quantitative analysis are presented here as mean ± SD. Paired‐sampled *t*‐tests were performed to compare the proposed approaches and the “standard” reconstruction in terms of septal blood‐myocardium sharpness, SNR of blood and myocardium and their CNR. The Wilcoxon signed‐rank test was performed on the categorical variables (i.e., subjective scores) to evaluate any differences between the reconstruction techniques. A *p*‐value <0.05 was considered significant.

## 3 RESULTS

### 3.1 Phantom study

**FIGURE 4:**
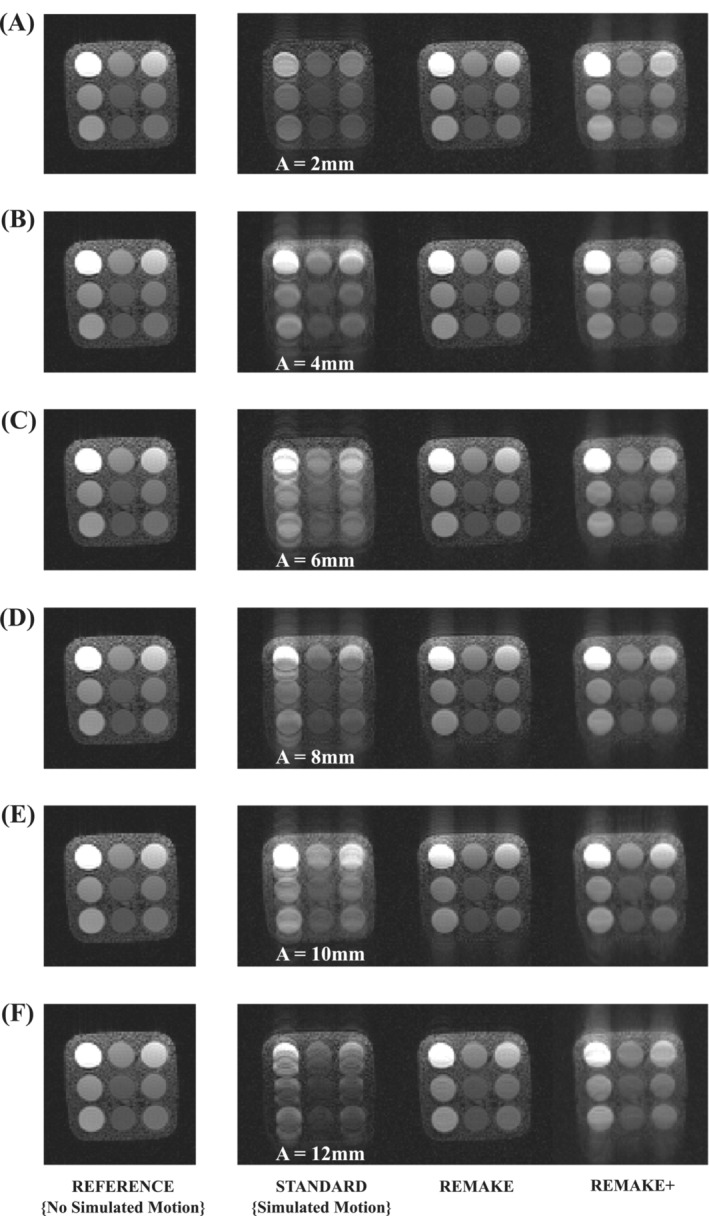
Phantom experiment demonstrating the benefit of REMAKE and REMAKE+ reconstructions using simulated motion corrupted data. Each row shows the reference image (no simulated motion) and the three reconstructions of simulated motion corrupted data (“standard”, REMAKE, and REMAKE+) for different respiratory motion amplitudes (defined from the variable A in Eq. 3): (A) A = 2 mm, (B) A = 4 mm, (C) A = 6 mm, (D) A = 8 mm, (E) A = 10 mm, and (F) A = 12 mm. REMAKE and REMAKE+ reconstructions provided substantial artifact reduction when compared to the “standard” reconstruction.

Representative images for each experiment with various motion amplitudes are shown in Figure 4. Severe motion artifacts are visible in all “standard” reconstructions and were substantially reduced with REMAKE and REMAKE+. Both REMAKE and REMAKE+ had lower RMSE (1.5 ± 0.4 and 1.0 ± 0.4) when compared to “standard” (2.2 ± 0.5, *p* < 0.01 for both), relative to the reference images. There were no differences in terms of SNR between REMAKE and “standard” (46.6 ± 0.6 vs. 47.3 ± 10.1, *p* = 0.9). REMAKE+ led to higher SNR (73.4 ± 4.1) than the “standard and REMAKE reconstructions (*p* < 0.01 for both). The accuracy (%) of correctly discarded segments across all experiments with the six different motion amplitudes was 89.3 ± 8.2, 94.5 ± 6.4, 87.7 ± 15.3, 91.9 ± 8.0, 90.9 ± 9.6 and 88.2 ± 14.9, respectively.

### 3.2 Patient study

**FIGURE 5:**
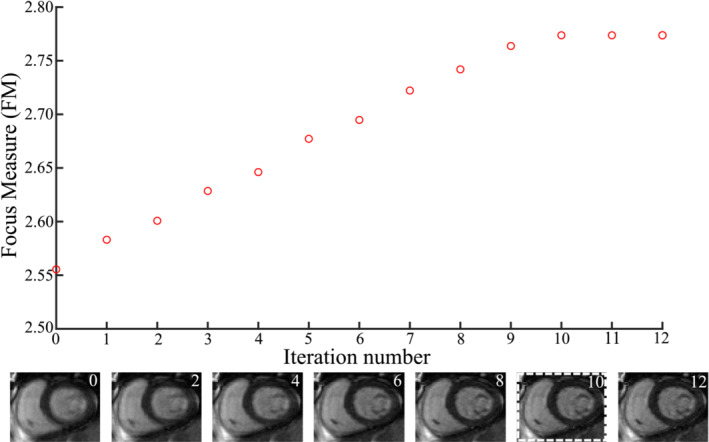
Evolution of focus measure as a function of iteration number (equals number of segments removed) for one slice and cardiac phase in one patient. The corresponding images at every other iteration are depicted to show image improvement with iterations. The focus measure begins to converge and drop off after 10 segments.

Figure 5 shows the evolution of the image focus and reconstructed images as a function of segments removed (i.e., at each iteration) in one example case. Improvement of image quality and image sharpness is observed with increasing iteration number. In the example shown, the focus measure converged after 10 iterations.

**FIGURE 6:**
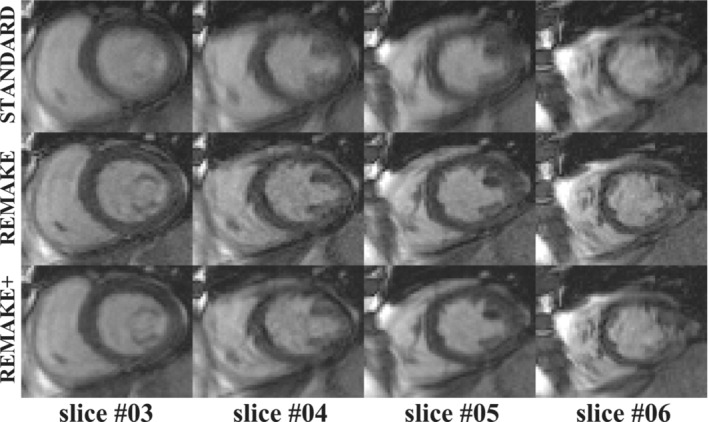
Case example where the “standard” reconstruction led to important motion artifacts. Reconstructions of four SAX slices are shown for one cardiac phase comparing the three reconstruction methods (“standard”, REMAKE, and REMAKE+). Improved image quality and image sharpness are observed for all slices in both REMAKE and REMAKE+ reconstructions relative to the “standard” reconstruction.

**FIGURE 7:**
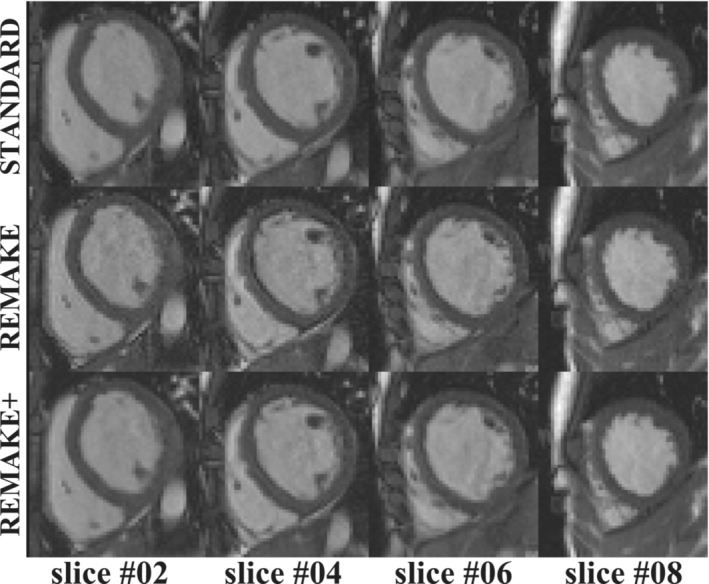
Case example where the “standard” reconstruction led to excellent image quality. Reconstructions of four SAX slices are shown for one cardiac phase comparing the three reconstruction methods (“standard”, REMAKE, and REMAKE+). All reconstructions resulted in excellent image quality and comparable sharpness. While some noise amplification can be observed using REMAKE, REMAKE+ avoided any noticeable noise amplification with respect to the “standard” reconstruction.

Example images reconstructed from two patients using the three reconstruction techniques are shown in Figures 6, 7. The animation of all slices of the cases presented in Figures 6, 7, showing all cardiac phases, is provided as Videos [Link], [Link]. Figure  6/Video  S1 provides a case example where the “standard” reconstruction presented blurring and motion artifacts. Improved image quality and image sharpness are observed for all slices in both REMAKE and REMAKE+ reconstructions relative to the “standard” reconstruction. REMAKE led to lower myocardial SNR, blood SNR and myocardial‐blood CNR (25/78/53) than the “standard” (36/104/68) and REMAKE+ (36/108/72) reconstructions. Figure 7/Video S2 provides a case example where the “standard” reconstruction led to excellent image quality. In this case, REMAKE and REMAKE+ also resulted in images of excellent quality. Some noise amplification can be observed using REMAKE, while REMAKE+ avoided any noticeable noise amplification with respect to the “standard” reconstruction. This is reflected in the quantification, where REMAKE led to lower myocardial SNR, blood SNR and myocardial‐blood CNR (29/92/63) than the “standard” (32/101/69) and REMAKE+ (40/126/87) reconstructions. REMAKE+ exceeded REMAKE in SNR and CNR.

**FIGURE 8:**
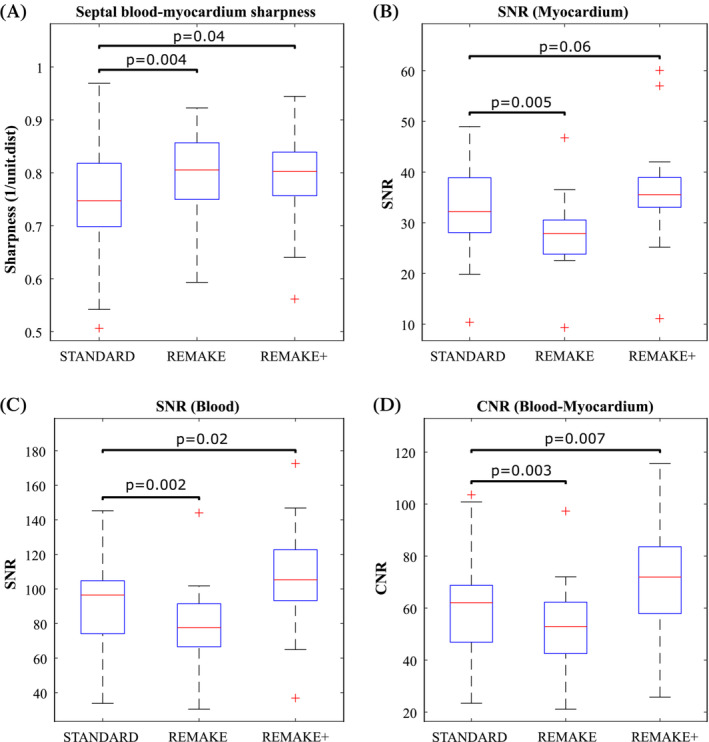
Quantitative analysis of sharpness, SNR, and CNR over all patients for the “standard”, REMAKE, and REMAKE+ reconstructions: (A) Septal blood‐myocardium sharpness. (B) Myocardial SNR. (C) LV blood pool SNR. (D) Blood‐myocardium CNR. REMAKE+ resulted in higher sharpness, SNR and CNR than “standard”.

Over all patients, septal blood‐myocardium sharpness increased significantly in REMAKE (0.79 ± 0.09) and REMAKE+ (0.79 ± 0.1) in comparison to “standard” (0.74 ± 0.12, *p* = 0.004 & *p* = 0.04, respectively) (Figure 8A). Blood SNR in “standard” (94 ± 30) was higher than in REMAKE (80 ± 25, *p* = 0.002) but lower than in REMAKE+ (105 ± 33, *p* = 0.02). Myocardial SNR in “standard” (33 ± 10) was higher than in REMAKE (28 ± 8, *p* = 0.005) and tended to be lower than in REMAKE+ (36 ± 12), although that difference did not reach statistical significance (*p* = 0.06) (Figures 8B, C). Similarly, myocardial‐blood CNR in “Standard” (61 ± 22) was higher than in REMAKE (53 ± 19, *p* = 0.003) and lower than in REMAKE+ (69 ± 24, *p* = 0.007) (Figure 8D).

**FIGURE 9:**
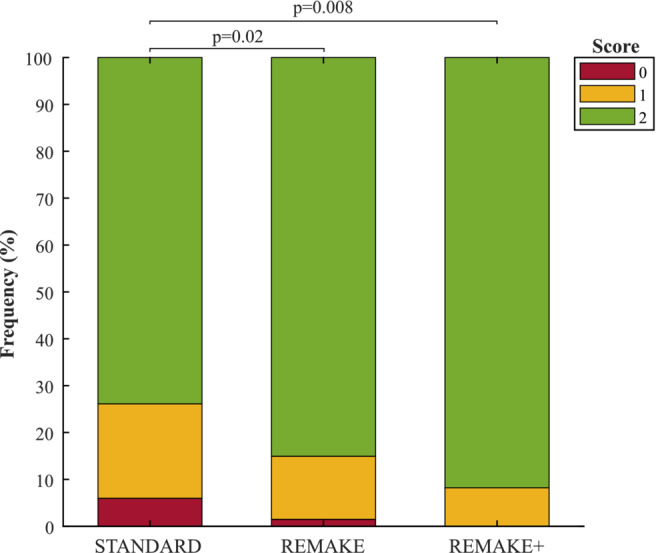
Qualitative image assessment. Scores: 0 = non‐diagnostic/severe motion artifacts, 1 = diagnostic (sub‐optimum image quality ‐ presence of motion artifacts/blurring not preventing interpretation/analysis of the images), 2 = diagnostic (excellent image quality ‐ no motion artifacts/no blurring/sharp myocardial edges). Subjective scores for REMAKE and REMAKE+ were significantly higher than “standard”.

Figure 9 shows that image quality scores obtained with REMAKE (1.8 ± 0.2) and REMAKE+ (1.9 ± 0.2) were higher than in “standard” (1.6 ± 0.4, *p* = 0.02 & *p* = 0.008, respectively). Furthermore, 94% and 99% of slices were of diagnostic value in “standard” and REMAKE, respectively, while 100% of slices were of diagnostic value in REMAKE+. Non‐diagnostic slices were observed in 4 patients (27%), 2 patients (14%) and none (0%) with “standard”, REMAKE, and REMAKE+, respectively.

## 4 DISCUSSION

In this study, we developed the REMAKE reconstruction method to detect and reject segments with significant contributions to blurring and motion artifacts from segmented cine data acquired with three signal averages. The proposed combined multi‐reconstruction approach (REMAKE+) with non‐rigid registration successfully provided improved SNR with respect to REMAKE alone. In‐vivo, both REMAKE and REMAKE+ resulted in improved image quality, myocardial sharpness, and rate of diagnostic quality images with respect to the standard image reconstruction.

REMAKE does not require any additional hardware or sequence modification, does not affect the temporal footprint of the sequence as the reconstruction is performed independently for each cardiac phase, and is fully automated. The overall scan time for a short‐axis stack was <4 min which is in a similar range as standard breath‐hold protocols, where rest times are needed between breath‐holds. REMAKE can be generalized, in theory, to any number of signal averages, and any sequence and trajectory. An example of an additional application will be in flow imaging, where free‐breathing acquisition with multiple averages can be used in patients unable to breath‐hold^7^ and may minimize the respiratory effects of breath‐holding on flow measurements.^41^, ^42^

The proposed reconstruction is computationally expensive (˜1 h for an entire SAX stack of cine images using a non‐optimized implementation in MATLAB). A substantial reduction of the computation time is necessary to facilitate online reconstruction and its integration into clinical routine. Graphics processing units (GPUs) have been successfully employed for MRI reconstruction^43^, ^44^ including iterative reconstruction of cardiac MRI data^45^ and can provide a substantial reduction of reconstruction time by a factor of up to 100–300.^46^ Furthermore, modern commercial MRI scanners are increasingly equipped with GPUs. In REMAKE/REMAKE+, the reconstruction process of the entire CINE stack is mostly applied independently to each slice and cardiac phase which offers a huge potential for parallelization and may be particularly well suited for a GPU implementation. Therefore, fast online reconstruction of REMAKE/REMAKE+ may be feasible using GPUs which may facilitate integration into clinical routine.

In the proposed reconstruction, the respiratory motion state of the final reconstruction is currently not controlled and depends on the initial condition (central k‐space segment selection). Noticeable changes or jumps due to respiratory motion between cardiac phases have not been observed in this study. The presence of varying respiratory motion states (i.e., jumps) between consecutive cardiac phases is unlikely to occur, as all cardiac phases from one given series are reconstructed using the k‐space central segments from the same NSA (which are acquired continuously within the same heartbeat).

It is, however, difficult to determine if consistent or different respiratory motion states between slices have been reconstructed for a given SAX stack cine series. Although no noticeable slice inconsistency was observed visually, which could, in theory, be possible. Since end‐expiration is the longest phase in the respiratory cycle,^47^ statistically, the majority of acquired k‐space segments would be acquired during end‐expiration, potentially resulting in segments at other phases in the respiratory cycle being discarded and promoting consistency between slices.

Motion artifacts such as blurring, ghosting, signal voids, and signal pileups will all contribute (either as an increase or decrease) to the final focus value. However, the quantification of their respective contributions to the focus measure is difficult to determine. Visual assessment of motion‐corrupted images in our study suggests blurring can be observed in the heart but also in abdominal organs and appears as the dominant artifact. This may explain the success of the employed focus measure.

In this study, segments acquired at different motion states were discarded. Although not applied for cine or dynamic imaging, methods have been proposed that apply motion compensation on k‐space data^48^ or k‐space segments acquired at different respiratory positions within a single free‐breathing 3D acquisition.^29^ Global motion parameters, such as affine deformation, were optimized for each, or a group of k‐space segments to improve the image sharpness of the heart. This strategy could also be combined with REMAKE in theory. The respiratory‐induced motion of the heart does not represent accurately the motion of surrounding tissue, particularly with the presence of static anatomical structures not subject to respiratory motion. By estimating transformations that prospectively correct for heart motion, surrounding static tissue, for example, may artificially be moved, distorting and inducing ghosting artifacts in the image. Furthermore, such an approach is also expected to further extend the computational time of the reconstruction process. Comparison of removal and motion correction of motion‐corrupted segments remains to be investigated in this context.

This study has some limitations. First, since this reconstruction was developed for patients unable to breath‐hold, this technique has not been compared to breath‐hold cine acquisitions, which are unsuitable in this patient population. However, it is important to note that breath‐hold cine is likely to outperform the proposed approach in patients with adequate breath‐holding capability. All in‐vivo analysis was based on SAX data. Cine imaging is also commonly performed in other orientations such as in long axis.^49^ Although the proposed reconstruction is expected to perform similarly in all orientations, further studies will be needed to confirm this hypothesis. Image reconstruction in this study was performed from raw data directly extracted from the scanner and did not include certain standard steps, such as phase correction, coil correction, or partial echo extrapolation. However, since this same reconstruction pipeline was applied to all three reconstruction methods to ensure a fair comparison, this should have had a very limited impact on the study. Finally, although the diagnostic value of images has been quantified, the reproducibility of other clinical metrics (such as myocardial segmentation and estimation of LV functional parameters) was not evaluated. Further studies will be needed to quantify the clinical benefit of this technique, which will require a much larger cohort given the relatively low rate of non‐diagnostic image quality observed with the standard technique.

## 5 CONCLUSIONS

A motion robust reconstruction technique based on the iterative rejection of k‐space segments was developed for retrospective correction of respiratory motion in multiple NSA, free‐breathing cine imaging. In comparison to standard signal average reconstruction, the proposed REMAKE and REMAKE+ techniques provide improved image sharpness, image quality, and rate of diagnostic quality images.

## CONFLICT OF INTEREST STATEMENT

Dr. Radhouene Neji is an employee of Siemens Healthcare and Dr. Ronald Mooiweer is seconded to Siemens Healthcare.

## Supporting information


**Table S1.** The 11 focus measures investigated and compared for REMAKE reconstructions of all 15 patient datasets.
**Figure S1.** Comparison of 11 focus measures for REMAKE. Reconstructions of the first cardiac phase of all slices spanning the heart in the short axis orientation using the 11 focus measures were performed on all 15 patients.
**Figure S2.** The fraction of segments (i.e. percentage of the total number of segments in a given slice or cardiac phase) removed was investigated across the patient population (N=15).

**Video S1.** Animation of all slices of the case example presented in Figure 6 showing all cardiac phases.

**Video S2.** Animation of all slices of the case example presented in Figure 7 showing all cardiac phases.
