# Supplementary material for: Retrospective motion correction through multi‐average k‐space data elimination (REMAKE) for free‐breathing cardiac cine imaging
Source: Magn Reson Med. 2023 Feb 10;89(6):2242–54. doi: 10.1002/mrm.29613 (PMC10952356; doi:10.1002/mrm.29613)
Supplement: Supplementary file 1 — Table S1. The 11 focus measures investigated and compared for REMAKE reconstructions of all 15 patient datasets. Figure S1. Comparison of 11 focus measures for REMAKE. Reconstructions of the first cardiac phase of all slices spanning the heart in the short axis orientation using the 11 focus measures were performed on all 15 patients. Figure S2. The fraction of segments (i.e. percentage of the total number of segments in a given slice or cardiac phase) removed was investigated across the patient population (N=15). [file MRM-89-2242-s002.docx]

**Supporting Information**

**In-vivo analysis of discarded segments**

**Supporting Information Figure S2** highlights the fraction of segments removed (i.e. percentage of the total number of segments in a given slice or cardiac phase) across a variety of tests. **Figure S3(A)** highlights the mean fraction of segments removed as measured across all cardiac phases and slices within the short-axis stack for each patient. The average fraction of segments removed, measured across all patients was 54±4%. The distribution of segments removed as a function of cardiac phase and slice with the short axis stack is highlighted in **Figures S3(B)** and **S3(C)**, respectively. The fraction of segments removed across cardiac phases appears to be more consistent (i.e. low variability is observed) for all patients. In contrast, the spread of segments removed varies more significantly across different slices within the short axis stack for all patients. This variability appears to not be related to slice location (as seen in **Figure S3(E)**), suggesting this may be due to the actual breathing pattern of the patient during the acquisition of a given slice. As shown in **Figure S3(D)**, a higher subjective score tended to be associated with a lower fraction of segment rejection (Median=59.4% (score=2) vs median=62.8% (score=1) and median=63.2% (score=0)), potentially related to an initial reconstruction of high quality/limited motion corrupted data.

| **Focus Measure** | **Description** |
| --- | --- |
| Brenner gradient (BREN) [1] | Gradient-based focus measure. |
| Energy of image gradient (GRAE) [2] | Gradient-based focus measure. |
| Gaussian derivative (GDER) [3] | Gradient-based focus measure. |
| Threshold absolute gradient (GRAT) [4] | Gradient-based focus measure. |
| Squared gradient (GRAS) [5] | Gradient-based focus measure. |
| Tenegrad (TENG) [6] | Gradient-based focus measure. |
| Tenegrad variance (TENV) [7] | Gradient-based focus measure. |
| Energy of Laplacian (LAPE) [2] | Laplacian-based focus measure. |
| Modified Laplacian (LAPM) [8] | Laplacian-based focus measure. |
| Diagonal Laplacian (LAPD) [9] | Laplacian-based focus measure. |
| Histogram Entropy (HISE) [6] | Statistical-based focus measure. |

**Supporting Information Table S1:** The 11 focus measures investigated and compared for REMAKE reconstructions of all 15 patient datasets.


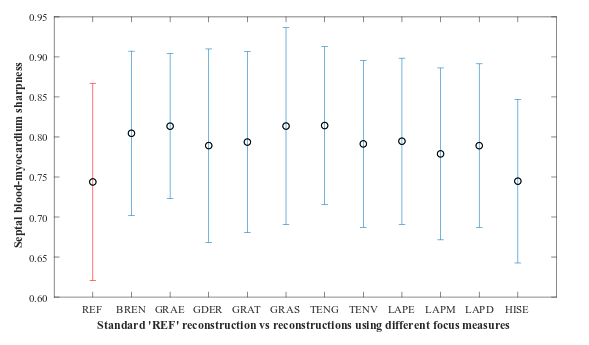
 **Supporting Information Figure S1:** Comparison of 11 focus measures for REMAKE. Reconstructions of the first cardiac phase of all slices spanning the heart in the short axis orientation using the 11 focus measures were performed on all 15 patients. The sharpness across the septal blood and myocardium boundary was determined quantitatively, for each reconstruction using each focus measure as described in Supporting Information Table S1. The result of which shows that the energy of image gradient (GRAE), squared gradient (GRAS) and Tenegrad (TENG) similarly outperform the other 8 other focus measures. The GRAE and GRAS focus measures were 3-5 times computationally faster at computing the focus of an image relative to the TENG measure and since the distribution of GRAE scores was less spread vs the GRAS scores (SD=0.09 vs SD=0.12), the energy of image gradient was chosen out of the three.

**
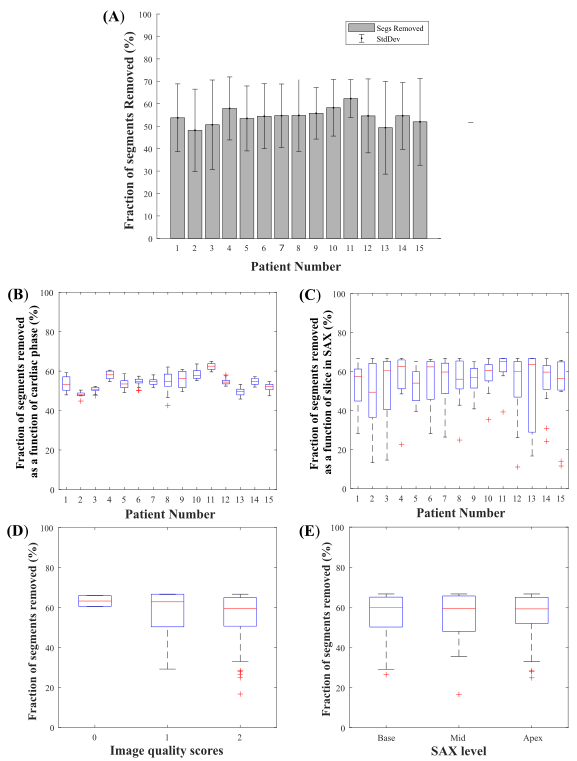
**

**Supporting Information Figure S2:** The fraction of segments (i.e. percentage of the total number of segments in a given slice or cardiac phase) removed was investigated across the patient population (N=15). **(A)** The mean fraction of segments removed as measured across all cardiac phases and slices within the short-axis stack. **(B)** The distribution of segments removed as a function of cardiac phase. **(C)** The distribution of segments removed as a function of slice within the short axis stack. **(D)** The distribution of segments removed for a given image with a subjective score (0, 1 or 2). **(E)** The distribution of segments removed for a given image at a specific region of the short-axis stack (Base, Mid or Apex).

**Supporting Information Video S1:** Animation of all slices of the case example presented in Figure 6 showing all cardiac phases.

**Supporting Information Video S2:** Animation of all slices of the case example presented in Figure 7 showing all cardiac phases.

**REFERENCES**

1. Brenner, J.F., et al., *An automated microscope for cytologic research.* J. Histochem. Cytochem, 1971. **24**: p. 100-111.

2. Subbarao, M., T. Choi, and A. Nikzad, *Focusing Techniques.* Optical Engineering, 1993. **32**(11): p. 2824-2836.

3. Geusebroek, J.-M., et al., *Robust autofocusing in microscopy.* Cytometry, 2000. **39**(1): p. 1-9.

4. Santos, A., et al., *Evaluation of autofocus functions in molecular cytogenetic analysis.* J Microsc, 1997. **188**(Pt 3): p. 264-72.

5. Shah, M.I., et al., *Identification of robust focus measure functions for the automated capturing of focused images from Ziehl-Neelsen stained sputum smear microscopy slide.* Cytometry A, 2017. **91**(8): p. 800-809.

6. Krotkov, E., *Focusing.* International Journal of Computer Vision, 1988. **1**(3): p. 223-237.

7. Pech-Pacheco, J.L., et al. *Diatom autofocusing in brightfield microscopy: a comparative study*. in *Proceedings 15th International Conference on Pattern Recognition. ICPR-2000*. 2000.

8. Nayar, S.K. and Y. Nakagawa, *Shape from focus.* IEEE Trans. Pattern Anal. Machine Intell., 1994. **16**: p. 824-831.

9. Thelen, A., et al., *Improvements in shape-from-focus for holographic reconstructions with regard to focus operators, neighborhood-size, and height value interpolation.* IEEE Trans Image Process, 2009. **18**: p. 151–157.
